# COVID-19 in people with HIV in the Netherlands

**DOI:** 10.1097/QAD.0000000000003597

**Published:** 2023-05-11

**Authors:** Ferdinand W.N.M. Wit, Peter Reiss, Bart Rijnders, Casper Rokx, Anna Roukens, Kees Brinkman, Marc van der Valk

**Affiliations:** a Stichting HIV Monitoring; b Amsterdam University Medical Centers, University of Amsterdam, Department of Infectious Diseases, Amsterdam Infection & Immunity Institute, Amsterdam; c Erasmus University Medical Center, Departments of Internal Medicine & Medical Microbiology, Rotterdam; d Leiden University Medical Center, Department of Internal Medicine, Leiden; e Onze Lieve Vrouwe Gasthuis, Department of Internal Medicine, Amsterdam, Netherlands.

**Keywords:** COVID-19, hospitalization, outcomes, SARS-CoV-2

## Abstract

We investigated occurrence of and risk factors for severe COVID-19 outcomes in people with HIV (PWH) in the Netherlands.

**Design:**

An ongoing prospective nationwide HIV cohort study.

**Methods:**

COVID-19 diagnoses and outcomes with other relevant medical information were prospectively collected from electronic medical records in all HIV treatment centers in the Netherlands, from the start of the COVID-19 epidemic until December 31, 2021. Risk factors for COVID-19 related hospitalization and death were investigated using multivariable logistic regression, including demographics, HIV-related factors, and comorbidities.

**Results:**

The cohort comprises 21 289 adult PWH, median age 51.2 years, 82% male, 70% were of Western origin, 12.0% were of sub-Saharan African and 12.6% Latin American/Caribbean origin, 96.8% had HIV-RNA less than 200 copies/ml, median CD4^+^ cell count 690 (IQR 510–908) cells/μl. Primary SARS-CoV-2 infections were registered in 2301 individuals, of whom 157 (6.8%) required hospitalization and 27 (1.2%) ICU admission. Mortality rates were 13 and 0.4% among hospitalized and nonhospitalized individuals, respectively. Independent risk factors for severe outcomes (COVID-19-related hospitalization and death) were higher age, having multiple comorbidities, a CD4^+^ cell count less than 200 cells/μl, uncontrolled HIV replication, and prior AIDS diagnosis. Migrants from sub-Saharan Africa, Latin America, and the Caribbean were at an increased risk of severe outcomes independently of other risk factors.

**Conclusion:**

In our national cohort of PWH, risk of severe COVID-19 outcomes was increased in individuals with uncontrolled HIV replication, low CD4^+^ cell count, and prior AIDS diagnosis, independently of general risk factors such as higher age, comorbidity burden and migrants originating from non-Western countries.

## Introduction

Between February 27, 2000, and December 31, 2021, an estimated cumulative 3.1 million individuals had become infected and 20 897 had died of COVID-19 in the Netherlands [1,2]. In the Netherlands, the SARS-CoV-2 vaccination program started in January 2021. At the start of the SARS-CoV-2 vaccination program, people with HIV (PWH) as a group were not prioritized; instead, initially, only the oldest PWH and those living in a nursing home were eligible. As of April 2021, all PWH became eligible for SARS-CoV-2 vaccination. National treatment guidelines for moderate and severe COVID-19 cases were continuously updated throughout the epidemic [3]. These guidelines did not consider PWH to be at a strongly increased risk for severe COVID-19.

Individuals however who are older, male, belong to non-Western ethnic groups, with lower socioeconomic status, and those with certain underlying conditions such as obesity, hypertension, renal dysfunction, diabetes mellitus, and cardiovascular disease, are at an increased risk for severe COVID-19, hospitalization, and death [4–6]. People with certain primary immunodeficiency syndromes, hematological malignancies, solid organ transplants, and people receiving immune-suppressive/immune-modulatory treatments likewise are at an increased risk of severe COVID-19 outcomes [7]. Many studies from general population COVID-19 cohorts in western countries have reported migrants and individuals belonging to non-Western ethnic groups to be at an increased risk of COVID-19 related hospitalization and death [8–15] with possible explanations including lower socioeconomic status and a higher prevalence (and severity) of comorbid conditions. In the general population of the Netherlands, people with a non-Western ethnicity (with the largest groups being of Dutch Antillean, Moroccan, Surinamese, Turkish, and Ghanaian descent) had a higher risk of COVID-19 hospitalization than those of Dutch ancestry [16]. In the Netherlands, the associations between the presence of comorbidities and risk of severe COVID-19-related outcomes have been reported to be stronger in some non-Western ethnic groups than in other groups [17]. Although data to answer the question if PWH are at an increased risk of severe COVID-19 may not be fully conclusive [18], most larger cohort studies have suggested an increased risk of severe outcomes [19–23] in PWH. A meta-analysis looking at the risk of COVID-19-related mortality in PWH, mainly including studies from North America and Europe, reported a pooled relative risk of 1.23 [24], very similar to the adjusted odds ratio of 1.29 reported by a large cohort study from the USA [25]. In PWH on antiretroviral therapy with well controlled HIV-replication and preserved CD4^+^ cell counts, underlying comorbidities and other general risk factors for severe COVID-19-related outcomes appear to play a larger role than HIV-related factors [26,27]. Importantly, whereas most of these studies adjusted their analyses for age, sex, ethnicity, and comorbidities, many of these were conducted as part of general COVID-19 population-based studies, and often did not have detailed data available on potentially relevant HIV-related parameters, such as use and type of antiretroviral therapy, plasma HIV-RNA levels, prior AIDS diagnoses, and current and nadir CD4^+^ cell counts. Therefore, it currently remains unclear which people with HIV in particular are at an increased risk of severe COVID-19-related outcomes. Of note, many of the risk factors for severe COVID-19 in the general population are more prevalent in PWH and whether a possibly increased risk in PWH is driven by differences in demographic characteristics, a high prevalence of non-HIV-related comorbidities, and/or HIV-related factors remains to be elucidated.

We report on the incidence of COVID-19 and risk factors for severe outcomes in the nationally representative adult population of PWH in the Netherlands using all available data collected up to December 31, 2021.

## Materials and methods

### Setting

The AIDS Therapy Evaluation in the Netherlands (ATHENA) national observational HIV cohort was established in 1998, following the introduction of combination antiretroviral therapy (cART) in the Netherlands in 1996 [28]. Since its inception in 2001, Stichting HIV monitoring (SHM) is managing the ATHENA cohort. SHM is funded by the Dutch government. In the Netherlands, all HIV care is provided in 24 designated HIV treatment centers, all of which are participating in the ATHENA cohort. In the HIV treatment centers, HIV care is provided in internal medicine and pediatric departments by infectious disease specialists. The Dutch guidelines for HIV care are based on international guidelines. All antiretroviral agents licensed in Europe are available in the Netherlands and can be used at the discretion of the treating physician. HIV care in the Netherlands is fully covered under basic health insurance, and also for undocumented HIV-positive migrants without health insurance HIV care and ART is provided free of charge. About 98% of PWH in care in the Netherlands have consented to participate in the ATHENA cohort, and 2% have opted-out. ATHENA is an open cohort, as people newly diagnosed with HIV continue to be enrolled into the cohort on entry into HIV care in one of the 24 HIV treatment centers. ATHENA prospectively collects relevant HIV and antiretroviral therapy (ART)-related data from the electronic health records (EHRs) in the HIV treatment centers. Table 1 provides details on the state of HIV care in the Netherlands.

**Table 1:** Characteristics of all ATHENA cohort participants and individuals diagnosed with COVID-19.

	All PWH	COVID-19, but not hospitalized	Hospitalized with COVID-19	*P* value group 2 vs. 3
*N*	21 289	2143	158	
Age (years)	51.2 (41.4–59.1)	48.8 (39.1–56.7)	58.1 (51.7–65.2)	<0.001
Male sex	82%	80%	77%	0.49
HIV transmission category				<0.001
MSM	64%	64%	42%	
Other men	18%	16%	35%	
Women	18%	20%	23%	
Region of origin				0.014
Western	70%	60%	51%	
Sub-Saharan Africa	12%	12%	20%	
Latin America/Caribbean	13%	16%	18%	
Other	5%	13%	10%	
Years known to be living with HIV	12.5 (7.2–18.6)	11.9 (6.6–17.7)	16.0 (9.6–22.5)	<0.001
On ART	98%	99%	99%	0.42
Current ART containing				
Tenofovir disoproxil	30%	30%	26%	0.24
Tenofovir alafenamide	43%	43%	45%	0.58
Abacavir	17%	15%	21%	0.042
NNRT inhibitor	31%	31%	30%	0.60
Protease inhibitor	16%	13%	23%	<0.001
Integrase inhibitor	57%	60%	59%	0.81
HIV viral load >200 cps/ml	3%	2%	7%	<0.001
Current CD4^+^ cell count (cells/μl)	690 (510–908)	710 (533–901)	605 (400–830)	<0.001
Nadir CD4^+^ cell count (cells/μl)	248 (119–380)	260 (130–400)	160 (60–270)	<0.001
Prior AIDS diagnosis	22%	18%	39%	<0.001
Comorbidities				
Obesity (BMI >30 kg/m^2^)	12%	14%	31%	<0.001
Diabetes mellitus type 2	5%	5%	17%	<0.001
Cardiovascular disease	4%	3%	9%	<0.001
Stroke	2%	2%	7%	<0.001
Hypertension (grade 2+ or on medication)	13%	12%	25%	<0.001
Non-AIDS-defining malignancy	4%	2%	5%	0.032
Chronic kidney disease (eGFR <30 ml/min)	1%	1%	3%	<0.001
Multimorbidity count				<0.001
0	69%	71%	38%	
1	23%	22%	36%	
2 or more	8%	7%	26%	

*N* (%) or median (IQR), as appropriate. ART, antiretroviral therapy; eGFR, estimated glomerular filtration rate; NNRT, nonnucleoside reverse transcriptase; Western includes native Dutch people, and migrants from Western Europa, North America, Japan, Australia, and New Zealand.

### Study design

ATHENA as of November 2020 uses automated electronic queries of EHRs in the HIV treatment centers to quickly identify new diagnoses of SARS-CoV-2 infection and prioritizes additional data collection regarding diagnosis, disease severity, hospitalizations, and outcomes of COVID-19 events. Even though the data collection process for COVID-19 events is prioritized, it should be noted that data collection does not happen in real time. Therefore, delays remain between the COVID-19 event having occurred, the information being recorded in the EHR in the HIV treatment centers, and the moment the data are captured in the ATHENA database and become available for analysis. In this analysis, we included COVID-19 events that occurred between February 2020, when the first COVID-19 cases were recorded in the Netherlands, up to December 31, 2021, with the data collection process continuing until July 2022.

### COVID-19 events

Data collection of COVID-19 events is based on the WHO ISARIC CRF [29], which is a standardized data collection tool, which aims to improve patient care and inform the public health response by facilitation COVID-19 research. The main focus of the COVID-19 data collection is on hospitalized patients, as individuals diagnosed with mild COVID-19 who are not admitted to hospital, only rarely have reliable detailed information documented in their HIV treatment center EHR for SHM to capture. SHM has not (yet) established linkage to other COVID-19 providers and cohorts/datasets, so direct comparisons with other patient populations currently cannot be made. Data on SARS-CoV-2 vaccination status were not available for all ATHENA participants.

### Outcome measures

For many cases, objective measures of COVID-19 disease severity could not be recorded by SHM, as these data were often not systematically recorded in the EHR of the HIV treatment center, especially for cases that were not hospitalized or cases that were hospitalized in another hospital than where they receive their HIV care. Therefore, the main outcome measures of this analysis are COVID-19 associated hospitalizations and death.

### Statistical analysis

Risk factors for COVID-19-related hospitalization and death were investigated using multivariable logistic regression, including relevant demographics (age, sex at birth, region of origin), established other risk factors (comorbidities), and HIV-related parameters (current and nadir CD4^+^ cell count categorized as 0–199, 200–499, and over 500 cells/μl; current plasma HIV-1 viral load above or below 200 copies/ml; having a prior diagnosis of an AIDS-defining condition; current use of ART; current use of individuals antiretroviral agents from the four commonly used drug classes with one of the most commonly used agent in each class being the reference group. Region of origin was categorized as Western (native Dutch people, and migrants from Western Europa, North America, Japan, Australia, and New Zealand); sub-Saharan African; Latin American/Caribbean; and other ethnicities. The final multivariable models were constructed using a stepwise forward model selection procedure, wherein the final model retained only variables that were significantly associated with the outcome. The presence of the following comorbidities and conditions known to increase the risk for severe COVID-19 were taken into account: cardiovascular disease (either myocardial infarction, coronary artery bypass grafting, coronary angioplasty or stenting, and carotid endarterectomy); stroke; non-AIDS-defining malignancies, excluding nonmelanoma skin cancers and premalignant lesions found at cervical/anal screening; chronic kidney disease (eGFR below 30 ml/min per 1.73 m^2^); diabetes mellitus (defined as having glycated hemoglobin levels above 52 mmol/mol and/or the use of antidiabetic medication); hypertension, defined as the use of antihypertensive drugs and/or measured grade 2 (or higher, 2018 ESC/ESH Guidelines on hypertension) hypertension with systolic pressure at or above 160 mmHg and/or diastolic pressure at or above 100 mmHg; and obesity (BMI over 30). The association between these comorbidities and the risk of severe COVID-19 were investigated both by entering them into the regression models separately, and as a multimorbidity covariate, that is, the sum of all seven abovementioned conditions. For the analyses of risk factors for COVID-19 related hospitalizations and mortality, we performed two sensitivity analyses. In the first sensitivity analysis, the multivariable regression analyses were limited to the participants who were diagnosed with COVID-19 in the prevaccination period to limit potential bias from differential uptake of SARS-CoV-2 vaccines. In the second sensitivity analysis, all ATHENA participants were included to limit potential bias from differential access to COVID-19 testing. All reported *P* values are two-sided, with *P* values below 0.05 considered statistically significant. All analyses were performed using SAS statistical software (version 9.4; SAS Institute, Cary, North Carolina, USA).

## Results

Between February 27, 2020, and December 31, 2021, 2301 primary SARS-CoV-2 infections were registered among 21 289 adult PWH: *n* = 2281 (99.1%) SARS-CoV-2 PCR-positive and an additional 20 (0.9%) SARS-CoV-2 PCR-negative but clinically strongly suspected cases (Table 1). An additional 264 possible SARS-CoV-2 infections were self-reported by individuals who had experienced mild symptoms possibly caused by SARS-CoV-2 infection but without PCR confirmation. These had mostly occurred in the early months of the epidemic in 2020 when SARS-CoV-2 testing was not yet widely available for mild cases. None of these possible SARS-CoV-2 infections resulted in hospitalization and are therefore not further considered in this report.

Of the 2301 individuals with a registered SARS-CoV-2 infection and considered for our analysis, 158 (6.9%) were hospitalized, with 27 (1.2%) requiring ICU admission. Of the remaining 2143 (93.2%) individuals, 50 (2.2%) did present with COVID-19 to an emergency room, but did not require hospitalization.

Those diagnosed with SARS-CoV-2 infection, but not hospitalized, were similar with regard to demographics, comorbidities, and HIV-related characteristics when compared with the total population of PWH in care in the Netherlands at the end of 2020, except for being substantially more likely to be born in a non-Western country (Table 1).

Those who were hospitalized for COVID-19 however were older, more likely to have acquired HIV through heterosexual contact (in men and to a lesser extent women), and more likely to be born in sub-Saharan Africa or Latin America and the Caribbean, when compared with the total population of PWH in care in the Netherlands at the end of 2020. Overall, men were not more likely to be hospitalized for COVID-19 than women, as the percentage of men among hospitalized patients (77.2%) was even somewhat lower than in the total population of PWH (81.9%). Each investigated comorbidity was more prevalent among those hospitalized compared with those not hospitalized for COVID-19, also resulting in a higher multimorbidity count in the hospitalized group (Table 1). The median duration of hospitalization was 6 (IQR 3–14) days. Individuals who were admitted to the ICU remained hospitalized for a median of 19 (12–34) days.

Regarding HIV-related characteristics, there were only minor differences between PWH who were diagnosed with but not hospitalized for COVID-19 and the total population of PWH, with the overwhelming majority being on ART, having a plasma HIV-1 viral load below 200 cps/ml, and high median CD4^+^ cell count well above 500 cells/μl. There were, however, notable differences between people diagnosed with COVID-19 who were hospitalized compared with those diagnosed with COVID-19 who were not hospitalized. Those who were hospitalized were on average 9.3 years older and had been living with HIV for 4.1 years longer. Furthermore, they had a lower median current (605 vs. 710 cells/μl, respectively) and nadir CD4^+^ cell count (160 vs. 260 cells/μl, respectively) and a higher prevalence of prior history of AIDS (39 vs. 18%, respectively) (Table 1).

**Fig. 1:**
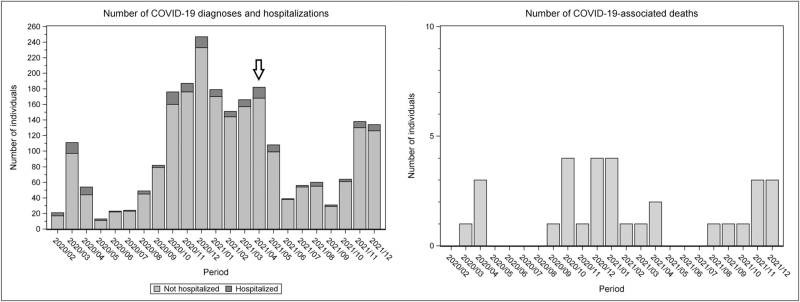
Incidence of COVID-19 diagnoses, hospitalizations, and deaths over calendar time.

Figure 1 shows the number of registered SARS-CoV-2 infections, hospitalizations for COVID-19, and COVID-19-related deaths during the study period. The peaks and troughs of the epidemic waves in PWH largely resemble those observed for the general population of the Netherlands [1,2].

Arrow denotes the month in which SARS-CoV-2 vaccination became available for all PWH in the Netherlands.

Risk factors for COVID-19-related hospitalization among PWH diagnosed with COVID-19, identified by multivariable logistic regression, included higher age, migrant status (with a higher risk in individuals originating from sub-Saharan Africa and to a lesser extent in Latin America and the Caribbean), and a higher number of concomitant comorbidities. In addition, having a current CD4^+^ cell count below 200 cells/μl, a last measured HIV viral load of more than 200 copies/ml, and a history of prior AIDS were each also independently associated with a higher odds of hospitalization (Table 2). None of the other demographic and HIV and ART-related parameters were independently associated with a higher risk of being hospitalized following a diagnosis of SARS-CoV-2 infection. In two sensitivity analyses, the first of which utilized data limited to the prevaccination period and the second included all ATHENA participants, yielded very similar results (Table 2). Supplemental Figure 1 shows the crude hospitalization rates per age group, CD4^+^ cell count category, and comorbidity count.

**Table 2:** Predictors of hospitalization among people with HIV who were diagnosed with COVID-19.

	Univariable analysis		Multivariable model		Sensitivity analysis 1 Multivariable model limited to prevaccination period		Sensitivity analysis 2 Model including all ATHENA participants	
Risk factor	Odds ratio (95% CI)	*P*	Odds ratio (95% CI)	*P*	Odds ratio (95%CI)	*P*	Odds ratio (95% CI)	*P*
Male sex	0.78 (0.53–1.15)	0.21						
Age (per 10 years increase)	1.90 (1.64–2.20)	<0.0001	1.71 (1.44–2.03)	<0.0001	1.60 (1.29–1.98)	<0.001	1.54 (1.33–1.80)	<0.001
Region of birth								
Western	-ref-		-ref-		-ref-		-ref-	
Sub-Saharan Africa	1.94 (1.24–3.04)	0.0036	2.06 (1.25–3.39)	0.0047	2.22 (1.21–4.10)	0.011	1.97 (1.23–3.13)	0.005
Latin America/Caribbean	1.41 (0.91–2.18)	0.12	1.31 (0.81–2.13)	0.28	1.08 (0.58–2.02)	0.81	2.10 (1.35–3.26)	0.001
Other	0.79 (0.43–1.44)	0.44	0.85 (0.46–1.60)	0.62	0.77 (0.35–1.72)	0.52	0.94 (0.52–1.70)	0.83
Number diagnosed comorbidities (per 1 more)	2.29 (1.91–2.76)	<0.0001	1.73 (1.40–2.13)	<0.0001	1.81 (1.39–2.36)	<0.001	1.57 (1.31–1.88)	<0.01
Current CD4^+^ cell count (cells/μl)								
0–199	5.60 (2.88–11.10)	<0.0001	3.53 (1.65–7.57)	0.0012	3.94 (1.52–10.2)	0.005	2.23 (1.16–4.28)	0.016
200–499	2.07 (1.43–3.00)	0.0001	1.47 (0.98–2.20)	0.062	1.73 (1.05–2.85)	0.32	1.24 (0.85–1.80)	0.26
500+	-ref-		-ref-		-ref-		-ref-	
Nadir CD4^+^ cell count (per 100 cells/μl increase)	0.72 (0.64–0.80)	<0.0001						
HIV viral load >200 copies/ml	2.41 (1.49–3.92)	0.0004	1.98 (1.13–3.47)	0.017	1.04 (0.45–2.37)	0.94	4.05 (2.40–6.85)	<0.001
Prior AIDS diagnosis	2.78 (1.97–3.93)	<0.0001	1.78 (1.16–2.72)	0.0010	2.18 (1.37–3.45)	0.001	1.62 (1.15–2.28)	0.006
Current use of ART								
NRTI^a^								
Tenofovir disoproxil	-ref-							
Tenofovir alafenamide	1.30 (0.86–1.97)	0.22						
Abacavir	1.63 (0.99–2.70)	0.058						
No NRTI	1.21 (0.67–2.16)	0.53						
NNRTI								
Efavirenz	-ref-							
Doravirine	0.43 (0.14–1.26)	0.12						
Rilpivirine	0.82 (0.35–1.96)	0.66						
Other	1.02 (0.47–2.22)	0.95						
No NNRTI	0.91 (4.8–1.73)	0.76						
Protease inhibitor (PI)								
Darunavir	-ref-							
Atazanavir	0.36 (0.046–2.73)	0.32						
No PI	0.50 (0.33–0.76)	0.0010						
Integrase inhibitor (INSTI)								
Dolutegravir	-ref-							
Bictegravir	1.06 (0.62–1.82)	0.82						
Other	1.02 (0.69–1.51)	0.19						
No INSTI	0.70 (0.41–1.20)	0.92						

95% CI, 95% confidence interval; ART, antiretroviral therapy; NNRTI, nonnucleoside reverse transcriptase inhibitor; NRTI, nucleoside-analogue reverse transcriptase inhibitor; ref, reference group. ^a^Tenofovir and abacavir were mostly used in combination with either emtricitabine or lamivudine; Western includes native Dutch people, and migrants from Western Europa, North America, Japan, Australia, and New Zealand.

In total, 31 (1.35%) out of the 2301 PWH diagnosed with SARS-CoV-2 infection were reported to have died as a direct result of COVID-19. The observed mortality in the various age groups was 0% [*n* = 0, odds ratio (OR) <0.001, *P* = 0.96] in 587 individuals aged 18–39 years, 0.2% (*n* = 1, OR 0.24, *P* = 0.19) in 592 individuals aged 40–49 years, 0.7% (*n* = 5, reference group) in 701 individuals aged 50–59 years, 3.1% (*n* = 10, OR 4.38, *P* = 0.0075) in 328 individuals aged 60–69 years, 13.0% (*n* = 10, OR 20.78, p<0.001) in 77 individuals aged 70–79 years, and 31.3% (*n* = 5, OR 63.27, *P* < 0.001) in 16 individuals aged over 80 years (Supplemental Figure 1). COVID-19-related mortality was 0.47% (10/2144) among those who had not been hospitalized, compared with 13.4% (21/157, odds ratio 33.0, *P* < 0.001) among those hospitalized for COVID-19, 37.0% (10/27, odds ratio 63.1, *P* < 0.001) among those admitted to the ICU. Of the nine individuals who died without having been hospitalized, eight were known to be living in a nursing home prior to being diagnosed with SARS-CoV-2 infection. The ninth individual was an unvaccinated 69-year-old Latin American man with a CD4^+^ cell count below 500 cells/μl with chronic renal failure due to HIV-related nephropathy.

Table 3 summarizes the demographics, HIV-related characteristics, and concomitantly diagnosed comorbidities of those who died of COVID-19 compared with those who recovered. The PWH who died of COVID-19 generally had poorer health prior to the onset of COVID-19, with a higher number of concomitantly diagnosed comorbidities and less favorable HIV-related parameters.

**Table 3:** Characteristics of individuals diagnosed with COVID-19 who died of COVID-19 compared with those who survived.

	Survived	Died of COVID-19
Number of individuals	2270	31
Age (years)	49.3 (39.6–57.0)	68.7 (60.9–78.2)
Male sex	80%	81%
HIV transmission category		
MSM	63%	45%
Other men	17%	36%
Women	20%	19%
Region of origin		
Western	59%	45%
Sub-Saharan Africa	12%	13%
Latin America/Caribbean	16%	32%
Other	13%	10%
Years known HIV-positive	12.1 (6.6–17.9)	22.1 (13.6–24.0)
On ART	99%	100%
HIV viral load >200 cps/ml	3%	3%
Current CD4^+^ cell count (cells/μl)	710 (529–900)	417 (316–789)
Nadir CD4^+^ cell count (cells/μl)	255 (130–390)	119 (62–220)
Prior AIDS diagnosis	19%	29%
Comorbidities		
Obesity (BMI >30)	15%	17%
Diabetes mellitus	5%	24%
Cardiovascular disease	3%	14%
Stroke	2%	28%
Hypertension (grade 2+ or on medication)	12%	55%
Non-AIDS-defining malignancy	3%	10%
Chronic kidney disease (eGFR <60 ml/min)	1%	21%
Multimorbidity count		
0	69%	17%
1	23%	31%
2	6%	28%
3	1%	14%
4 or more	1%	10%

*N* (%) or median (IQR), as appropriate. Western includes native Dutch people, and migrants from Western Europa, North America, Japan, Australia, and New Zealand. eGFR, estimated glomerular filtration rate.

Because of the low number of COVID-19 related deaths, statistical power to formally explore risk factors using multivariable regression analysis was limited. Exploratory multivariable logistic regression models showed that independent risk factors for COVID-19 related mortality were higher age, being of Latin American origin, having a higher number of concomitantly diagnosed comorbidities, having a current CD4^+^ cell count below 500 cells/μl with an even higher risk when the CD4^+^ cell count was below 200 cells/μl (Table 4). In two sensitivity analyses, the first of which utilized data limited to the prevaccination period and the second included all ATHENA participants, yielded very similar results (Table 4). Supplemental Figure 1 shows the crude mortality rates per age group, CD4^+^ cell count category, and comorbidity count.

**Table 4:** Independent predictors of mortality among people with HIV who were diagnosed with COVID-19.

	Multivariable model		Sensitivity analysis 1 Model limited to prevaccine period		Sensitivity analysis 2 Model including all ATHENA participants	
Risk factor	Odds ratio (95% CI)	*P*	Odds ratio (95% CI)	*P*	Odds ratio (95% CI)	*P*
Age (per 10 years increase)	5.01 (3.18–8.17)	<0.001	6.23 (3.14–12.3)	<0.001	4.03 (2.71–6.01)	<0.001
Region of birth						
Western	-ref		-ref-		-ref-	
Sub-Saharan Africa	−2.96 (0.72–12.1)	0.13	1.69 (0.17–16.5)	0.65	3.33 (0.89–12.4)	0.073
Latin America/Caribbean	3.32 (1.19–9.21)	0.021	4.48 (1.12–18.0)	0.034	6.7 (2.78–16.2)	<0.001
Other	0.66 (0.13–3.39)	0.61	0.66 (0.06–8.03)	0.75	1.19 (0.27–5.31)	0.82
Number of concomitantly diagnosed comorbidities (per one comorbidity increase)	2.11 (1.40–3.19)	<0.001	2.11 (1.40–3.18)	<0.001	1.93 (1.40–2.67)	<0.001
Current CD4^+^ cell count						
0–199	6.48 (1.22–34.54)	0.029	12.7 (1.83–88.1)	0.010	4.54 (1.07–19.4)	0.041
200–499	2.80 (1.15–6.84)	0.024	4.14 (1.16–14.7)	0.028	2.49 (1.13–5.48)	0.024
500+	-ref-		-ref-		-ref-	

Western includes native Dutch people, and migrants from Western Europa, North America, Japan, Australia, and New Zealand. 95% CI, 95% confidence interval; ref, reference group.

We attempted to investigate possible differential associations between ethnicity and severity of COVID-19 for various comorbidities, but were limited by the low number of individuals diagnosed with each comorbidity. Only obesity, diabetes mellitus, and hypertension were sufficiently prevalent to allow for an exploratory analysis into hospitalizations for COVID-19, which consistently showed that migrants from sub-Saharan Africa diagnosed with one of these conditions were substantially more likely to be hospitalized for COVID-19 compared with individuals from the other migrant groups and the native Dutch diagnosed with the same conditions (data not shown). The number of COVID-19-related deaths in our cohort were too low to allow even for an exploratory analysis of a possible interaction between ethnicity and comorbidities.

## Discussion

Our analyses confirm that risk factors of severe COVID-19 outcomes in the general population also apply to PWH: older age, the presence of (multiple) comorbidities, belonging to a non-Western migrant population increase the risk of hospitalization and/or death. The observed hospitalization rates and mortality in PWH diagnosed with COVID-19 were very low in those aged below 50 years, but quickly increased in the older age strata. Independent of these general risk factors, having a low current CD4^+^ cell count, and to a lesser extent uncontrolled HIV replication and a prior AIDS diagnosis were also identified as risk factors. We did not observe an apparent protective effect of the concomitant use of tenofovir disoproxil, integrase inhibitors, nor of any other commonly used antiretroviral agent, as has been reported by other cohorts [20,21,30–33]. Other Western COVID-19 cohorts of PWH found similar patterns of risk factors for severe COVID-19 outcomes [25,34–41].

The observed hospitalization rate was 6.8% in all PWH diagnosed with COVID-19, and 1.2% were admitted to the ICU. The observed mortality rate in hospitalized individuals was 13.4%, and 31.3% for those admitted to the ICU. Mortality in PWH diagnosed with COVID-19 who were not hospitalized was very low (0.47%). Both the hospitalization and mortality rates likely represent an overestimation given that most cases of asymptomatic SARS-CoV-2 infection will have gone undiagnosed, especially in the beginning of the epidemic when testing was restricted to severely ill individuals [42]. Furthermore, eight of the nine PWH who were recorded as having died of COVID-19 without having been hospitalized, were already in poor health, and living in nursing homes.

In our study, migrants born in sub-Saharan Africa or Latin America and the Caribbean were at an increased risk of hospitalization and COVID-19-related mortality independent of age, comorbidities, and HIV-related parameters. However, these findings should be interpreted with caution because of the limited number of events available for analysis, and the possibility of residual confounding. Migrant populations in the Netherlands have been shown to be at a greater risk of SARS-CoV-2 acquisition compared with the general population [43]. They might be at an increased risk of acquiring SARS-CoV-2 infection related to a higher chance of having lower socioeconomic status, which is associated with more crowded and multigenerational housing conditions, higher residential neighborhood population density, and being employed in front-line jobs wherein SARS-CoV-2 exposure is more likely [43]. In addition, people with a migrant background with mild COVID-19 symptoms might have been less willing to be tested and/or had encountered more barriers to access testing, potentially further increasing the estimates for the risk of serious outcomes in these populations [44]. However, non-Western migrants remained at an increased risk for severe COVID-19 outcomes in sensitivity analyses in which all ATHENA cohort participants were included in the denominator.

An additional factor that might further contribute to the observed higher risk of severe outcomes in PWH from non-Western migrant groups compared with PWH from the general Dutch population and migrants from Western countries, could be a reduced willingness to be vaccinated against SARS-CoV-2 [45–48]. However, it should be noted that most of the observed COVID-19-related mortality has occurred before PWH became eligible for our national SARS-CoV-2 vaccination program, and hence, a lower vaccination uptake could at best only partially explain the increased risk of hospitalization and mortality in migrants. Furthermore, sensitivity analyses confirmed that in the prevaccine period, migrant populations from non-Western countries were also at an increased risk for severe COVID-19 outcomes independent from HIV-related and general risk factors.

This study has several strengths and limitations. The ATHENA cohort is a national HIV cohort in which 98% of all PWH in care in the Netherlands is included, making our findings representative for the Dutch population of people with HIV. The ATHENA cohort prospectively collects data on socio-demographics, general health status, comorbidities, and HIV and ART-related parameters, enabling us to investigate and identify the independent contributions of both general and HIV-related risk factors for severe COVID-19 outcomes. A limitation of our study is that we had incomplete data on the COVID-19 treatments administered to PWH hospitalized with COVID-19. However, we have no indication that PWH admitted for COVID-19 were less likely to receive guideline-recommended COVID-19 treatments. Furthermore, we had incomplete SARS-CoV-2 vaccination data, limiting our ability to assess the role of vaccine hesitancy in explaining the increased risk for severe COVID-19 outcomes in non-Western migrant populations. However, sensitivity analyses limited to the prevaccination period showed similar results. Other limitations are the lack of a matched general population comparison group, and lack of adjustment for some socioeconomic variables, such as household income, occupational exposure, household composition, which precluded us from investigating if differences in such parameters between ethnic groups could explain the observed increased risk for severe COVID-19 outcomes in non-Western migrant populations.

### Conclusion

In the Dutch population of PWH, we observed a low incidence of severe COVID-19 outcomes, very similar to what was observed in other Westerns cohorts of PWH. A major strength of our analysis is that we were able to account for both general and HIV-specific risk factors for severe COVID-19. We found that in PWH on ART with well controlled HIV infection and preserved CD4^+^ cell counts, underlying comorbidities and other general risk factors for severe COVID-19-related outcomes play a larger role than HIV-related factors. Although in Western countries the risk of severe COVID-19 is slightly increased in the population of PWH as a whole compared with the general population, the increased risk for severe COVID-19 is not distributed equally throughout the population of PWH, but is instead increased in older people, in those with (multiple) comorbidities, in non-Western minority migrant groups, and in the small sub-group of those with less favorable HIV-related parameters. To illustrate this point, in the 707 PWH diagnosed with COVID-19 who were below the age of 50, who had a CD4^+^ cell count over 500 cells/μl, and who had no comorbidities, just 1.7% was hospitalized and none died of COVID-19. In many populations of PWH, comorbidities are more prevalent than in the general population, either because of a higher prevalence of general risk factors for these comorbidities but possibly also because of direct effects of HIV itself as well as (prior) exposure to severe immune deficiency and ART-related toxicities.

## Acknowledgements

The ATHENA study group: **Amsterdam UMC, AMC site, Amsterdam:***HIV-treating physicians*: M. van der Valk∗, S.E. Geerlings, A. Goorhuis, V.C. Harris, J.W. Hovius, B. Lempkes, F.J.B. Nellen, T. van der Poll, J.M. Prins, V. Spoorenberg, M. van Vugt, W.J. Wiersinga, F.W.M.N. Wit. *HIV nurse consultants*: C. Bruins, J. van Eden, I.J. Hylkema-van den Bout, F.J.J. Pijnappel, S.Y. Smalhout, A.M. Weijsenfeld. *HIV clinical virologists/chemists:* N.K.T. Back, B. Berkhout, M.T.E. Cornelissen, R. van Houdt, M. Jonges, S. Jurriaans, C.J. Schinkel, K.C. Wolthers, H.L. Zaaijer. **Amsterdam UMC, VUmc site, Amsterdam:***HIV-treating physicians*: E.J.G. Peters∗, M.A. van Agtmael, R.S. Autar, M. Bomers, K.C.E. Sigaloff. *HIV nurse consultants*: M. Heitmuller, L.M. Laan. *HIV clinical virologists/chemists*: N.K.T. Back, B. Berkhout, M.T.E. Cornelissen, R. van Houdt, M. Jonges, S. Jurriaans, C.J. Schinkel, K.C. Wolthers, H.L. Zaaijer. **Admiraal De Ruyter Ziekenhuis, Goes:***HIV treating physicians*: M. van den Berge∗, A. Stegeman. *HIV nurse consultants*: S. Baas, L. Hage de Looff. *HIV clinical virologists/chemists*: A. van Arkel, J. Stohr, B. Wintermans. **Catharina Ziekenhuis, Eindhoven:***HIV treating physicians*: M.J.H. Pronk∗, H.S.M. Ammerlaan. *HIV nurse consultants*: E.S. de Munnik. *HIV clinical virologists/chemists*: B. Deiman, A.R. Jansz, V. Scharnhorst, J. Tjhie, M.C.A. Wegdam. **DC Klinieken Lairesse – Hiv Focus Centrum, Amsterdam:***HIV treating physicians*: M. van der Valk∗, A. van Eeden, E. Hoornenborg, J. Nellen. *HIV nurse consultants*: W. Alers, L.J.M. Elsenburg, H. Nobel. *HIV clinical virologists/chemists*: C.J. Schinkel. **ETZ (Elisabeth-TweeSteden Ziekenhuis), Tilburg:***HIV treating physicians*: M.E.E. van Kasteren∗, M.A.H. Berrevoets, A.E. Brouwer. *HIV nurse specialist*: B.A.F.M. de Kruijf-van de Wiel. *HIV nurse consultants*: A. Adams, M. Pawels-van Rijkevoorsel. *HIV data collection*: B.A.F.M. de Kruijf-van de Wiel. *HIV clinical virologists/chemists*: A.G.M. Buiting, J.L. Murck. **Erasmus MC, Rotterdam:***HIV treating physicians*: C. Rokx∗, A.A. Anas, H.I. Bax, E.C.M. van Gorp, M. de Mendonça Melo, E. van Nood, J.L. Nouwen, B.J.A. Rijnders, C.A.M. Schurink, L. Slobbe, T.E.M.S. de Vries-Sluijs. *HIV nurse consultants*: N. Bassant, J.E.A. van Beek, M. Vriesde, L.M. van Zonneveld. *HIV data collection*: J. de Groot. *HIV clinical virologists/chemists*: J.J.A. van Kampen, M.P.G Koopmans, J.C. Rahamat-Langendoen. **Flevoziekenhuis, Almere:***HIV-treating physicians*: J. Branger∗, R.A. Douma. *HIV nurse consultant*: A.S. Cents-Bosma, C.J.H.M. Duijf-van de Ven. **HagaZiekenhuis, Den Haag:***HIV treating physicians*: E.F. Schippers∗, C. van Nieuwkoop. *HIV nurse consultants*: J. Geilings, S. van Winden. *HIV data collection*: G. van der Hut. *HIV clinical virologists/chemists*: N.D. van Burgel. **HMC (Haaglanden Medisch Centrum), Den Haag:***HIV-treating physicians*: E.M.S. Leyten∗, L.B.S. Gelinck, F. Mollema. *HIV nurse consultants*: G.S. Wildenbeest. *HIV clinical virologists/chemists*: T. Nguyen. **Isala, Zwolle:***HIV treating physicians*: P.H.P. Groeneveld∗, J.W. Bouwhuis, A.J.J. Lammers. *HIV nurse consultants*: A.G.W. van Hulzen, S. Kraan, M.S.M. Kruiper. *HIV data collection*: G.L. van der Bliek, P.C.J. Bor. *HIV clinical virologists/chemists*: S.B. Debast, G.H.J. Wagenvoort. **Leids Universitair Medisch Centrum, Leiden:***HIV-treating physicians*: A.H.E. Roukens∗, M.G.J. de Boer, H. Jolink, M.M.C. Lambregts, H. Scheper. *HIV nurse consultants*: W. Dorama, N. van Holten. *HIV clinical virologists/chemists*: E.C.J. Claas, E. Wessels. **Maasstad Ziekenhuis, Rotterdam:***HIV-treating physicians*: J.G. den Hollander∗, R. El Moussaoui, K. Pogany. *HIV nurse consultants*: C.J. Brouwer, D. Heida-Peters, E. Mulder, J.V. Smit, D. Struik-Kalkman. *HIV data collection*: T. van Niekerk. HIV clinical virologists/chemists: O. Pontesilli, C. van Tienen. **Maastricht UMC+, Maastricht:***HIV-treating physicians*: S.H. Lowe∗, A.M.L. Oude Lashof, D. Posthouwer, M.E. van Wolfswinkel. *HIV nurse consultants*: R.P. Ackens, K. Burgers, M. Elasri, J. Schippers. *HIV clinical virologists/chemists*: T.R.A. Havenith, M. van Loo. **Medisch Centrum Leeuwarden, Leeuwarden:***HIV-treating physicians*: M.G.A. van Vonderen∗, L.M. Kampschreur. *HIV nurse consultants*: M.C. van Broekhuizen, S, Faber. *HIV clinical virologists/chemists*: A. Al Moujahid. **Medisch Spectrum Twente, Enschede:***HIV treating physicians*: G.J. Kootstra∗, C.E. Delsing. *HIV nurse consultants*: M. van der Burg-van de Plas, L. Scheiberlich. **Noordwest Ziekenhuisgroep, Alkmaar:***HIV treating physicians*: W. Kortmann∗, G. van Twillert∗, R. Renckens, J. Wagenaar. *HIV nurse consultants & HIV data collection*: D. Ruiter-Pronk, F.A. van Truijen-Oud. *HIV clinical virologists/chemists*: J.W.T. Cohen Stuart, M. Hoogewerf, W. Rozemeijer, J.C. Sinnige. **OLVG, Amsterdam:***HIV-treating physicians*: K. Brinkman∗, G.E.L. van den Berk, K.D. Lettinga, M. de Regt, W.E.M. Schouten, J.E. Stalenhoef, J. Veenstra, S.M.E. Vrouenraets. *HIV nurse consultants*: H. Blaauw, G.F. Geerders, M.J. Kleene, M. Knapen, M. Kok, I.B. van der Meché, A.J.M. Toonen, S. Wijnands, E. Wttewaal. *HIV clinical virologists*: D. Kwa, T.J.W. van de Laar. **Radboudumc, Nijmegen:***HIV treating physicians*: R. van Crevel∗, K. van Aerde, A.S.M. Dofferhoff, S.S.V. Henriet, H.J.M. ter Hofstede, J. Hoogerwerf, O. Richel. *HIV nurse consultants*: M. Albers, K.J.T. Grintjes-Huisman, M. de Haan, M. Marneef. *HIV clinical virologists/chemists*: M. McCall. *HIV clinical pharmacology consultant*: D. Burger. **Rijnstate, Arnhem: *HIV treating physicians:* E.H. Gisolf∗, M. Claassen, R.J. Hassing,.*****HIV nurse** consultants:* G. ter Beest, P.H.M. van Bentum, M. Gelling, Y. Neijland. *HIV clinical virologists/chemists:* C.M.A. Swanink, M. Klein Velderman. **Spaarne Gasthuis, Haarlem:***HIV-treating physicians*: S.F.L. van Lelyveld∗, R. Soetekouw. *HIV nurse consultants*: L.M.M. van der Prijt, J. van der Swaluw. *HIV clinical virologists/chemists*: J.S. Kalpoe, A. Wagemakers, A. Vahidnia. **Medisch Centrum Jan van Goyen, Amsterdam:** HIV treating physicians: F.N. Lauw, D.W.M. Verhagen. *HIV nurse consultants*: M. van Wijk. **Universitair Medisch Centrum Groningen, Groningen:***HIV treating physicians*: W.F.W. Bierman∗, M. Bakker, R.A. van Bentum, M.A. van den Boomgaard, J. Kleinnijenhuis, E. Kloeze, A. Middel, D.F. Postma, H.M. Schenk, Y. Stienstra, M. Wouthuyzen-Bakker. *HIV nurse consultants*: A. Boonstra, H. de Jonge, M.M.M. Maerman, D.A. de Weerd. *HIV clinical virologists/chemists*: K.J. van Eije, M. Knoester, C.C. van Leer-Buter, H.G.M. Niesters. **Universitair Medisch Centrum Utrecht, Utrecht:***HIV-treating physicians*: T.Mudrikova∗, R.E. Barth, A.H.W. Bruns, P.M. Ellerbroek, M.P.M. Hensgens, J.J. Oosterheert, E.M. Schadd, A. Verbon, B.J. van Welzen. *HIV nurse consultants*: H. Berends, B.M.G. Griffioen-van Santen, I. de Kroon. *HIV clinical virologists/chemists*: F.M. Verduyn Lunel, A.M.J. Wensing. **Coordinating center:***Board of directors*: M. van der Valk, S. Zaheri. *HIV data analysis:* A.C. Boyd, D.O. Bezemer, A.I. van Sighem, C. Smit, F.W.M.N. Wit. *Data HIV data management and quality control:* M.M.J. Hillebregt, T.J. Woudstra, T. Rutkens. *HIV data monitoring*: D. Bergsma, N.M. Brétin, K.J. Lelivelt, L. van de Sande, K.M. Visser, S.T. van der Vliet. *HIV data collection:* F. Paling, L.G.M. de Groot-Berndsen, M. van den Akker, R. Alexander, Y. Bakker, A. El Berkaoui, M. Bezemer-Goedhart, E.A. Djoechro, M. Groters, L.E. Koster, C.R.E. Lodewijk, E.G.A. Lucas, L. Munjishvili, B.M. Peeck, C.M.J. Ree, R. Regtop, A.F. van Rijk, Y.M.C. Ruijs-Tiggelman, P.P. Schnörr, M.J.C. Schoorl, E.M Tuijn, D.P. Veenenberg, E.C.M Witte. *Patient registration*: D. Bergsma, N.M. Brétin, Y.M.C. Ruijs-Tiggelman.

∗ denotes site coordinating physician

The authors received no specific funding for this work. The ATHENA cohort is supported by a grant from the Dutch Ministry of Health, Welfare and Sport through the Centre for Infectious Disease Control of the National Institute for Public Health and the Environment. Funding was not linked to any individual author. The funder had no role in study design, data collection and analysis, decision to publish, or preparation of the manuscript.

### Conflicts of interest

F.W. has received fees from ViiV for participation on advisory boards; P.R. through his institution has received independent scientific grant support from Gilead Sciences, Janssen Pharmaceuticals Inc, Merck & Co and ViiV Healthcare, and has served on scientific advisory boards for Gilead Sciences, ViiV Healthcare, and Merck & Co honoraria for which were all paid to his institution; B.R. has received research grants from Gilead, research grants from Merck Sharp and Dohme and honoraria from Jansen-Cilag, BMS, Pfizer, and ViiV; C.R. has received fees for advisory boards and unrestricted research grants from Gilead Sciences and ViiV; A.R. has disclosed no conflicts of interest; K.B. has disclosed no conflicts of interest; MvdV through his institution has received fees for advisory boards and unrestricted research grants from Gilead Sciences, Merck, and ViiV.

## Supplementary Material

Supplemental Digital Content
