# Supplementary material for: COVID-19 in people with HIV in the Netherlands
Source: AIDS. 2023 May 11;37(11):1671–81. doi: 10.1097/QAD.0000000000003597 (PMC10399951; doi:10.1097/QAD.0000000000003597)
Supplement: Supplemental Digital Content [file aids-37-1671-s001.docx]

**Supplemental** **Figure 1**: Proportions of COVID-19-related hospitalization and mortality by age group, CD4 cell count category, and co-morbidity count.


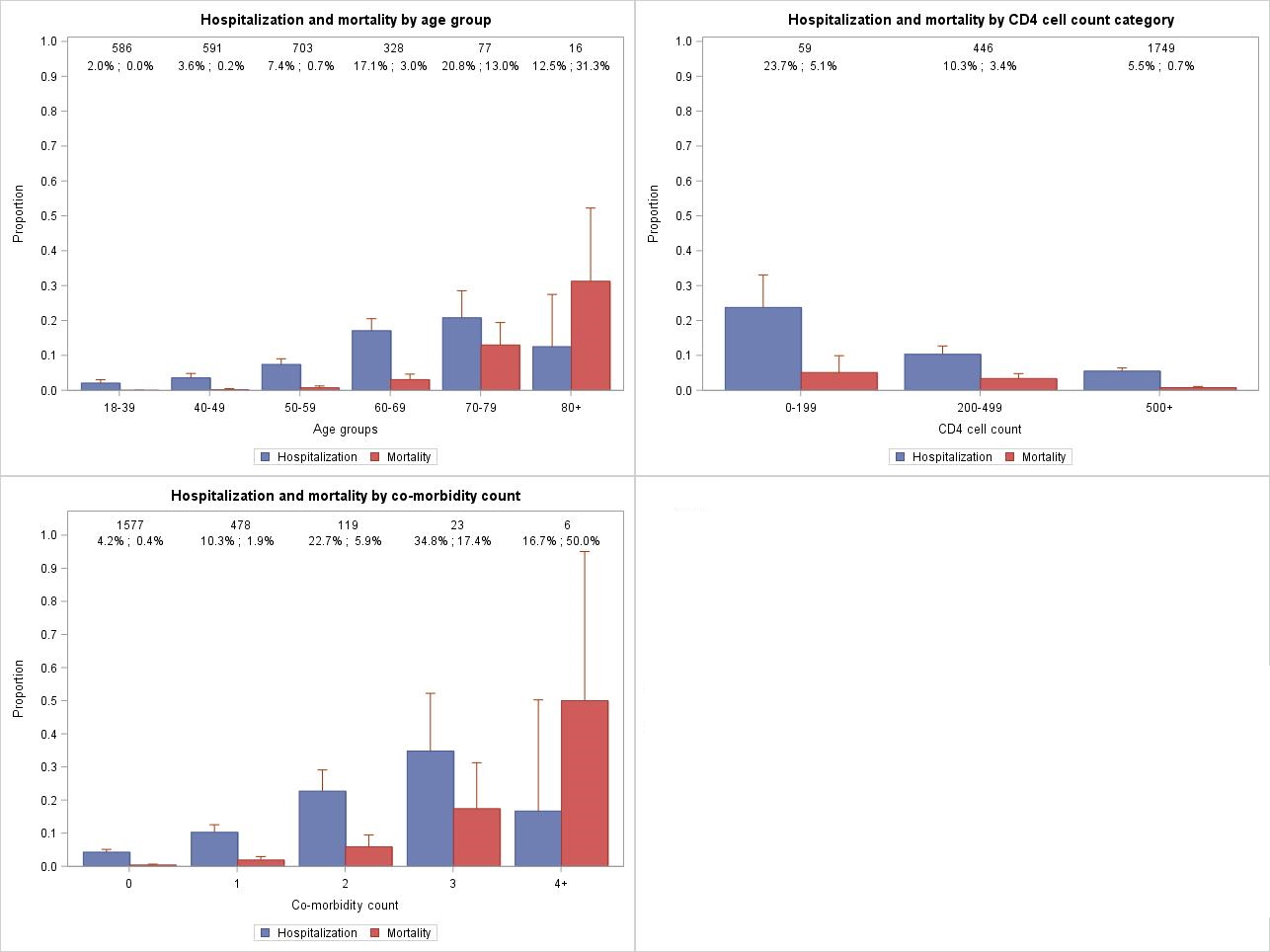


Figure legend: The numbers at the top of the panels denote the number of individuals (top row) and the percentage of hospitalized and deceased individuals (bottom row) in each category.
